# Fade In, Fade Out: Do Shifts in Visual Perspective Predict the Consistency of Real-World Memories?

**DOI:** 10.1177/09567976231180588

**Published:** 2023-07-13

**Authors:** Victoria Wardell, Taylyn Jameson, Oliver J. R. Bontkes, M. Lindy Le, Tz-yu Duan, Peggy L. St. Jacques, Christopher R. Madan, Daniela J. Palombo

**Affiliations:** 1 Department of Psychology, University of British Columbia; 2 Department of Psychology, University of Alberta; 3 School of Psychology, University of Nottingham

**Keywords:** autobiographical interview, autobiographical memory, memory consistency, visual perspective, open data

## Abstract

Memories of our personal past are not exact accounts of what occurred. Instead, memory reconstructs the past in adaptive—though not always faithful—ways. Using a naturalistic design, we asked how the visual perspective adopted in the mind’s eye when recalling the past—namely, an “own eyes” versus “observer” perspective—relates to the stability of autobiographical memories. We hypothesized that changes in visual perspective over time would predict poorer consistency of memories. Young adults (*N* = 178) rated the phenomenology of and freely recalled self-selected memories of everyday events at two time points (10 weeks apart). Multilevel linear modeling revealed, as expected, that greater shifts in visual perspective over time predicted lower memory consistency, particularly for emotional details. Our results offer insight into the factors that predict the fidelity of memories for everyday events. Moreover, our results may elucidate new metrics that are useful in interpreting eyewitness testimony or experiences relayed in clinical contexts.

Memories of our past, that is, autobiographical memories, allow us to recall what we have done and where we have been (Conway, 2005). Far from providing a stable record, however, memories are malleable. Prominent theories suggest that memories are constructed in order to facilitate navigating the present moment, often at the expense of recalling exact accounts of what happened (Conway & Pleydell-Pearce, 2000; Schacter et al., 2011). However, it is important that memories maintain some degree of accuracy so that we can learn from the past in meaningful ways (Conway & Loveday, 2015). If memories were too vulnerable to change, they would no longer provide us with useful information. To the extent that we rely on memories to be accurate in personal and societal (e.g., eyewitness testimony) realms, it is important to understand what factors impact an autobiographical memory’s vulnerability to change.

Autobiographical memory allows us to mentally travel to the past, evoking imagery, sensations, or emotions that can recreate the subjective experience of the original event (Rubin, 2006; Tulving, 1985). This sense of reexperiencing has been associated with the richness of the visual imagery elicited within the mind’s eye when remembering (Zaman & Russell, 2022). Such imagery requires a *visual perspective* from which to picture the event (Nigro & Neisser, 1983; Rubin & Umanath, 2015). For decades, researchers have puzzled over visual perspective. Despite experiencing our lives from our own egocentric point of view, memories can be pictured from one’s “own” perspective or an “observer’s” perspective—we can watch ourselves move through past events as if watching an actor on stage (Iriye & St. Jacques, 2020; Nigro & Neisser, 1983; Robinson & Swanson, 1993). (Although it has been speculated that situations involving self-evaluation, [Nigro & Neisser, 1983], or dissociation, [Bergouignan et al., 2022], might give rise to observer perspective as a memory is being formed.) These perspectives are not mutually exclusive; a memory for a single event can shift between own and observer perspectives over time and even over the course of a single recall (Rice & Rubin, 2009; St. Jacques et al., 2017).

**Statement of Relevance**

For many people, the act of remembering may make it feel like we can replay past experiences as if they were a video in our mind’s eye. Unlike a video, however, memories are malleable—often changing with each viewing. The mental imagery associated with a memory can manifest from varied vantage points; we can watch the event from our own eyes or take on a different perspective, watching ourselves moving through our past. In a sample of university students, we asked whether the point of view one adopts when remembering real-world events relates to the consistency of memories over time. We found that greater shifts in visual perspective predicted lower memory consistency, specifically for emotional content. The malleability of memory provides us with the capacity to play with past events, twisting and turning them in our mind. By adopting a new perspective, we may remember details differently, but in exchange we gain the opportunity to see our lives from another point of view.

In spite of its enigmatic quality, the significance of visual perspective is well recognized in disparate domains of psychology, including cognitive, social, and clinical science. In the domain of cognitive science, shifts in perspective have been linked with characteristics of memory, both in the subjective experience of remembering, such as the emotional intensity of the memory (Sekiguchi & Nonaka, 2014), and the details recalled (Akhtar et al., 2017; King et al., 2022), indicating that visual perspective is one indicator of memory malleability. Here, we asked whether changes in perspective predict the *consistency* with which voluntary autobiographical memories are recalled over time: Does a change in visual perspective confer a distortion of voluntarily recalled details of past events?

Such a speculation was advanced almost 40 years ago by Nigro and Neisser (1983). Although surprisingly little evidence has materialized since, some findings lend initial credence to the idea that perspective is linked to changes in memory. Compared to adopting an own perspective, recalling events from an observer’s perspective is associated with less detailed memories (Berntsen & Rubin, 2006; D’Argembeau et al., 2003; Sutin & Robins, 2010; Vella & Moulds, 2014) and less vivid memories (Berntsen & Rubin, 2006; Butler et al., 2016; Williams & Moulds, 2008). Compared to memories from an own persepective, memories from an observer’s perspective contain fewer sensory and affective details, although other types of details, such as physical appearance or spatial relationships, do not seem to systematically differ as a function of perspective (Bagri & Jones, 2009; King et al., 2022; McIsaac & Eich, 2002, 2004; Piolino et al., 2006). However, these studies do not address the issue of accuracy, insofar as these data show that memories from an observer perspective are less rich, but not necessarily less faithful, than memories from an own perspective.

More central to Nigro and Neisser’s (1983) proposal, one laboratory study provides a link between visual perspective change and memory *fidelity*: Marcotti and St. Jacques (2018) manipulated visual perspectives by asking participants to recall a staged event from either an own or an observer perspective—an approach that allowed the researchers to corroborate the accuracy of the recall. Intentionally shifting memories from own to observer perspective reduced memory accuracy, an effect driven by vividness. Other innovative approaches have been adopted to elucidate the relationship between perspective and accuracy, including reviewing photographs of staged events from different perspectives (Marcotti & St. Jacques, 2022) and manipulating perspective at the time of encoding via virtual reality (Iriye & St. Jacques, 2021). Still, in these studies, change in perspective was an externally imposed task, as opposed to a gradual, internally driven process. It remains unclear whether naturally occurring shifts in visual perspective are associated with changes in the fidelity of autobiographical memories.

Accordingly, we assessed how own and observer perspectives correlate with the consistency of autobiographical memories. We addressed our question through the lens of time, which creates naturalistic conditions to observe whether changes in perspective are associated with changes in memory consistency: Time exerts a major influence on memory, both in accuracy (Armson et al., 2017; Shapira & Pansky, 2019) and vividness (Cooper et al., 2019; Rice & Rubin, 2009). However, we do not merely forget details over time; we embellish memories with new information (Schacter, 2022). As a memory ages, it is less likely to maintain a faithful representation of what was encoded. Fittingly, memories tend to shift away from own and toward observer perspectives over time (Butler et al., 2016; King et al., 2022; Rice & Rubin, 2009)—though perspectives can shift in opposite directions, even if less frequent (McCarroll, 2017). Here, we tracked memories twice over 10 weeks. This design allowed us to test the hypothesis that natural changes in visual perspectives over time for voluntary memories would be associated with reduced consistency of real-world memories for everyday experiences. Although consistency cannot be considered synonymous with accuracy, it is a useful real-world proxy for the fidelity of memories that are otherwise unverifiable.

## Open Practices Statement

Deidentified data for this experiment, along with a codebook and data-analysis scripts, have been made publicly available via OSF and can be accessed at https://osf.io/hmt9a/. The design and analysis plans for the experiment were not preregistered. Materials are available on request from the corresponding author.

## Method

### Participants

Students from the University of British Columbia participated in this two-session online study in exchange for course credit. To be included in the analyses, participants had to complete both sessions, pass all attention checks embedded throughout the study (three in Session 1 and one in Session 2), and provide valid event recalls at both sessions. We defined a valid recall as being an event (a) that occurred within the last 3 weeks at the time of the participant’s first session, (b) that was remembered at the second session, and (c) for which participants gave narratives at both sessions that reflected remembering (e.g., the events could not be copy-and-pasted text or random keyboard entries).

After recruiting the maximum number of participants permitted by the participant pool, we had a total of 357 participants who completed the first session, although 16 of them failed attention checks and were excluded from further analysis. Of the 341 remaining participants, 192 returned to complete Session 2 (all of them passed the Session 2 attention check). Fourteen participants did not provide valid memory data and were excluded, leaving a final sample of 178 participants (age: *M* = 20.66 years, *SD* = 2.56; 84.3% women, 14.0% men, and 1.7% gender diverse). This study was approved by the local ethics committee of the University of British Columbia.

### Procedure

#### Session 1

Participants completed Session 1 online via the survey platform Qualtrics. After providing informed consent, demographic information, and health histories, participants were asked to select six everyday events (three control memories; detailed below) from their past that had occurred 1 to 14 days ago (e.g., “within the last two weeks, not including today”) and that they would be comfortable discussing. We requested that the events not be mundane, traumatic, or involve substance use but be distinct episodes that they could bring to mind (see the Supplemental Material available online). A 2-week interval was selected in order to capitalize on changes in memory observed soon after encoding (see Bauer, 2015) while providing a period reasonably long enough for participants to identify unique neutral events. Given the COVID-19 pandemic restrictions affecting this sample of students, we further asked that the events selected not include virtual coursework or virtual videoconferencing events (e.g., Zoom), as such instances might be difficult to differentiate from one another at the time of the second session. Participants were asked to provide a title and date for each event, which, unbeknownst to them, would be used to cue the events at Session 2.

After selecting the six events, participants were asked to self-report the visual perspective of their memories in a randomized order. We provided participants with a definition of both “own” and “observer” visual perspectives (see the Supplemental Material). Participants were then asked to rate the degree to which they pictured their memory of the event from both an own and observer perspective on separate scales (Rice & Rubin, 2009). Participants continued to rate their events on additional phenomenological characteristics, including memory vividness and emotionality of the event (for a complete list of ratings, see Table S1 in the Supplemental Material). Participants then answered, in one sentence, “please describe what made this event unique to you, that is, a distinct detail or occurrence from this event that makes it stand out in your mind” to serve as an additional cue in Session 2 if the memory title alone was not effective (see Session 2 description below). Coding was embedded into the survey to randomly select three of the six events that each participant provided to be recalled in a written narrative. The remaining three events were not recalled and served as control events to ensure that changes in phenomenological ratings of recalled events were not unduly influenced by virtue of recalling the memory for our study. Participants were asked to type out all the details they could remember about the three randomly selected events to be recalled. Participants were provided with an example memory to read to ensure they understood the types of details we were requesting them to provide (see the Supplemental Material). They then recalled each event one at a time. Participants were unable to progress in the survey until they had provided a minimum of 1,200 characters for their event. This was done to ensure task adherence and to encourage participants to provide all the details they could.

After providing recalls for three events, participants completed a battery of questionnaires for ancillary hypotheses to be tested outside of this article (see the Supplemental Material).

#### Session 2

Participants completed the second session approximately 10 weeks later. Participants were provided with the event title and date that they had provided in the first session for all six of their original events in a randomized order and were asked to indicate whether they remembered the event. If they indicated that they did, they proceeded to rerate the event on the same phenomenological ratings as Session 1. If they indicated that they did not remember the event, they were shown their response to the question “What made this event unique to you?” from Session 1 to use as a cue. Participants were asked to indicate whether they now recognized the event after seeing the cue. Participants then proceeded to the ratings, regardless of whether the event was remembered. Although events noted as not being remembered were not analyzed, ratings were still collected to ensure that participants did not indicate not remembering their event simply to speed through the study.

**Fig. 1.**
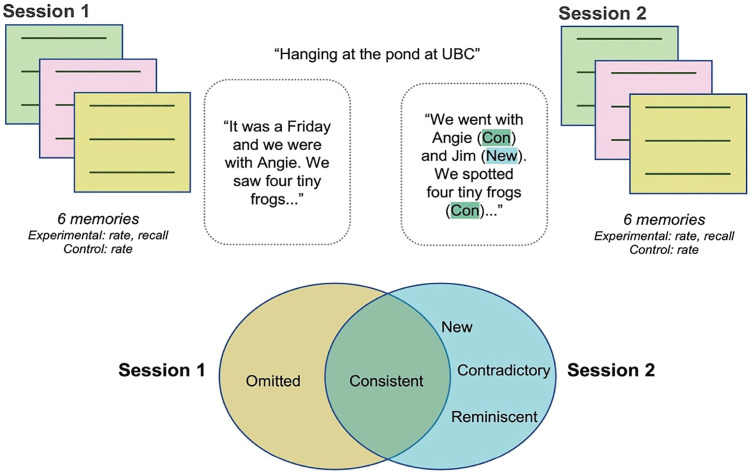
Overview of study design and Autobiographical Interview Consistency Supplement (AI-CONS) scoring. Participants recalled memories during two sessions approximately 10 weeks apart. Session 2 memories were scored for consistency of episodic details and could contain consistent (Con), new, contradictory, or reminiscent details. Session 1 memories were scored for omitted episodic details (i.e., details that were in Session 1 but not in Session 2). See Table 1 for definitions. See the Supplemental Material available online for a representative scoring example from our data.

Participants were then asked to type out all the details they could remember for the three events they had recalled during Session 1 in a random order. The same instructions and example memory were used to direct participants toward recalling as many details as possible. As in Session 1, each event was recalled, one at a time, and a minimum of 1,200 characters was required (see Fig. 1 for overview of study design).

Following recall, a second battery of questionnaires were administered for ancillary hypotheses to be tested outside of this article (see the Supplemental Material).

### Data processing

With 178 participants recalling three events each, there was an initial dataset of 534 events. However, prior to analyzing the data, we excluded 28 individual events for being outside of our date range. Although participants were instructed to provide events from within the last 2 weeks at Session 1, the accepted interval was increased to 3 weeks in order to preserve as much data as possible while maintaining recollections recent enough to capture changes in memory between sessions. A further 16 events were excluded because participants indicated that they could not remember the event at Session 2 despite the event title and one-sentence cue. Finally, 20 additional events were excluded because the typed recall did not reflect remembering (i.e., random key entries [four events], no internal details in the narrative [four events], or the recall provided at Session 2 was for the wrong event [12 events]). After excluding these events, we had a final dataset of 470 events. All 178 participants had at least one valid event recall that was included in analyses. Specifically, 10 participants had only one event included in analyses, 44 participants had two events, and the remaining 124 had all three events retained for analyses.

The written recalls of events from both sessions were scored according to the Autobiographical Interview (AI) scoring procedure (Levine et al., 2002). This procedure identifies the types of details produced during autobiographical memory recall. A detail is defined as any piece of information (such as an occurrence, observation, or thought) and is often associated with a grammatical clause. For example, “I found my mask in my car” would be scored as two details, one for “I found my mask” and one for “in my car.” In the AI procedure, details are categorized as internal and external. Internal details encompass any episodic detail that refers directly to the event being recalled, whereas external details encompass any detail that does not directly refer to the event being recalled. Because details unrelated or tangential to the event being recalled, such as information about other events (e.g., “just like the last time we went to the beach”) or semantic knowledge (e.g., “I like rocky beaches more than sandy beaches”), are not inherent to the accuracy of the recall, we considered details related to the specific episode only (i.e., internal details). Internal AI details were further divided into detail categories: event (i.e., what happened, who was there), perceptual (i.e., sensations and percepts), emotion/thoughts (i.e., emotions and thoughts), place (i.e., location), and time (i.e., temporal setting), in accordance with the AI protocol (Levine et al., 2002; Wardell, Esposito et al., 2021).

After event recalls were scored for AI detail types, the episodic details of corresponding transcripts between sessions were compared for their consistency using a novel procedure developed in our lab, which we call the AI-Consistency Supplement (AI-CONS; for similar approaches, see Dev et al., 2022; Odinot et al., 2013; Orbach et al., 2012). Episodic details in transcripts from Session 2 were identified as either consistent, contradictory, reminiscent, or new in relation to the details provided in transcripts from Session 1. Following Marcotti and St. Jacques’s (2018) conservative scoring scheme, in our study, we reserved the “consistency” category only for episodic details that nearly precisely matched the corresponding Session 1 detail. Furthermore, Session 1 transcript details not included at Session 2 were scored as “omitted” (see Table 1). For an example of a scored recall, see the Supplemental Material. A strength of our approach is that it allowed us to examine consistency across the canonical detail categories used in the AI protocol. Such an approach (i.e., combining the AI with consistency scoring) has not, to our knowledge, been employed in research on autobiographical memory. Using this technique, we can illuminate (a) what types of details are most and least consistent over time^1^ and (b) which consistent detail types, if any, are associated with visual perspective.

**Table 1.** Consistency of Episodic Details Scored in Narrative Recalls

AI-CONS detail type	Description	Example	
		Session 1 Transcript	Session 2 Transcript
Consistent	Detail was in both the Session 1 and Session 2 transcripts.	I met up with my friend.	I met my friend.
Contradictory	Detail in Session 2 transcript contradicted detail in Session 1 transcript.	It was so dark in the cave.	It was really bright in the cave.
Reminiscent	Detail in Session 2 transcript was reminiscent of detail in Session 1 transcript.	I was kind of embarrassed.	I felt so ashamed.
New	Detail in Session 2 transcript was not in Session 1 transcript.	—	It was late at night.
Omitted	Detail in Session 1 transcript was not in Session 2 transcript.	I was at my house.	—

Note: AI-CONS = Autobiographical Interview Consistency Supplement.

Six experimenters scored the data. As preparation, all six experimenters demonstrated reliable scoring of the AI by scoring memory narratives previously analyzed by the curators of the procedure. Four of these scorers went on to score transcripts for AI details. The remaining two scorers were further trained to score memories for consistency using the AI-CONS procedure. We confirmed the reliability of scoring across experimenters by asking all four of those who were conducting the AI procedure to score a subset of 10% of the memories and by asking both AI-CONS scorers to score a separate 10% of the memories. Intraclass correlation (ICC) analyses on these subsets of memories confirmed excellent agreement between scorers on internal AI details (α = .97) and AI-CONS consistency details (α = .94; see Table S3 in the Supplemental Material for ICCs of all detail types).

### Data analysis

To explore our main research question, namely, how shifts in visual perspectives relate to the consistency of episodic details in autobiographical memories over time, we first calculated shifts in visual perspective using absolute values of difference scores. Specifically, shifts in visual perspective between Session 1 and Session 2 were calculated by subtracting Session 1 ratings from Session 2 ratings. Absolute values were then calculated, with higher numbers indicating greater shifts and zero indicating that the rating did not change. We opted to use absolute values a priori on the assumption that any change in perspective should result in a change in consistency, not just shifts from an own to an observer perspective. Next, memory consistency was calculated as the proportion of consistent episodic details provided at Session 2 out of the total number of episodic details provided at Session 2. On the basis of our scoring scheme, we predicted a negative correlation between change in visual perspective and consistency.

We employed multilevel linear modeling (MLM) to examine the relationship between shifts in visual perspectives and memory consistency. MLM was selected because of its flexibility in modeling fixed and random effects. Participants and events were both treated as random effects, which allowed us to (a) account for our within-participants design and (b) consider memory-level as opposed to participant-level effects. That is, instead of aggregating across memories to reflect average tendencies of individual participants, we were able to examine each individual memory (see Devitt et al., 2017, for similar logic). Shifts in visual perspectives were treated as fixed effects and used as predictors in our hierarchical model with proportion of consistent details as our outcome variable. MLM analyses were run using the *R* package *lme4* (Bates et al., 2015).

## Results

### Descriptive statistics

#### General

Recalled events ranged from 1 to 19 days old at Session 1 (*M* = 7.08, *SD* = 4.20) and 73 to 105 days old at Session 2 (*M* = 84.44, *SD* = 5.15); days between sessions ranged from 71 to 93 (*M* = 77.36, *SD* = 3.15). Ratings of phenomenological characteristics showed that the memories selected were, on average, of midrange importance and uniqueness at both sessions, indicating that we were successful in capturing everyday but not overly mundane experiences (see Table S4 in the Supplemental Material). Critically, phenomenological characteristics of recalled events did not differ from rated events that were not recalled (i.e., control events) in Session 2 (see Table S5 in the Supplemental Material). This indicates that recalling events for our study per se did not alter their phenomenology. MLM analyses revealed that subjective ratings of memory vividness (β = −0.22, *p* < .001; *R*^2^ = .39, 95% confidence interval [CI] = [.34, .43]) and episodic (internal) details recalled (β = −0.25, *p* < .001; *R*^2^ = .49, 95% CI = [.45, .54]) decreased between sessions, showing that the data in our paradigm behaved in expected ways (i.e., memory fading), based on prior work, and further that our test-retest time frame was appropriate for assessing changes in memories. Together, these patterns in the data show that our paradigm elicited the appropriate types of memories to address our research question.

#### Visual perspective

Consistent with past research showing decreases in own and increases in observer visual perspectives over time, MLM analysis revealed that, overall, own perspective ratings tended to be lower at Session 2 than Session 1 (β = −0.18, *p* < .001; *R*^2^ = .36, 95% CI = [.31, .41]), whereas observer perspective ratings tended to be higher (β = 0.10, *p* < .001; *R*^2^ = .35, 95% CI = [.30, .40]; see Fig. S1 in the Supplemental Material). Still, shifts in perspectives for individual memories were not uniform: Descriptively, own perspective ratings at Session 2 decreased for 45.5% of events, 32.6% showed no change, and 21.9% increased. For observer perspectives, 26.8% of ratings at Session 2 decreased, 30.2% showed no change, and 43.0% increased. Furthermore, own and observer perspectives were negatively associated at Session 1 (β = −0.74, *p* < .001; *R*^2^ = .64, 95% CI = [.59, .69]) and Session 2 (β = −0.70, *p* < .001; *R*^2^ = .69, 95% CI = [.65, .74]), indicating that the two constructs are related but not redundant (see Rice & Rubin, 2009). These data show that (a) our design choice, namely, to place sessions approximately 10 weeks apart, successfully elicited sufficient changes in visual perspective across memories; (b) over time, both own and observer visual perspectives naturally shift up and down; and (c) separate analysis of own and observer visual perspectives is warranted.

#### Consistency

**Fig. 2.**
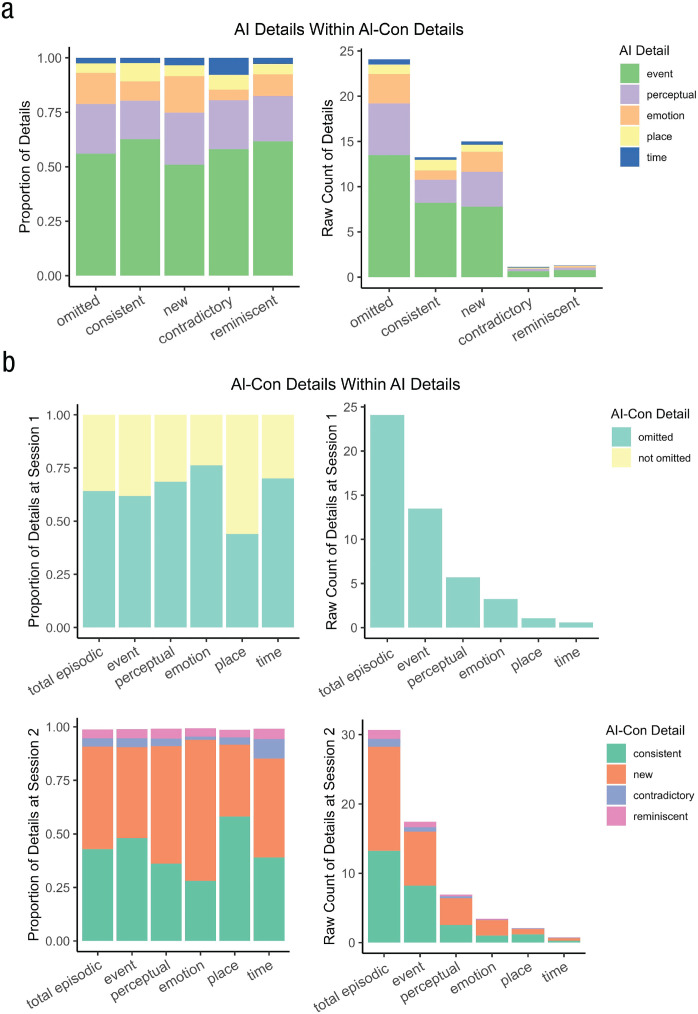
Memory consistency over time, given as both proportion of details (left column) and raw count of details (right column). (a) Average distribution of Autobiographical Interview (AI) details within each AI Consistency Supplement (AI-CONS) detail category. (b) Average distribution of AI-CONS details within each AI detail category, separately for Session 1 (top row) and Session 2 (bottom row). Because consistent, new, contradictory, and reminiscent details were scored in Session 2, the proportion of these AI-CONS details were calculated with AI details identified in Session 2. Because omitted details were scored in Session 1, the proportion of omitted details were calculated with AI details identified in Session 1.

Measured as the total number of consistent details divided by total episodic details at Session 2, consistency had a mean proportion of .43 (*SD* = .17). Two event recalls at Session 2 had no consistent internal details. However, these memories were quality-checked to confirm that participants had indeed recalled the same event at both sessions. Hence, as shown in Figure 2, overall, consistency was not very high, with a large spread across participants. Still, we note that the number of contradictory details was fairly low; memories were inconsistent not because of contradictions but because participants provided a lot of new information that was not recalled at Session 1 (and left out a lot of details provided at Session 1; i.e., errors of commission and omission; see Fig. 2).

### Main analyses

Visual inspection of density and quantile-quantile plots indicated that residuals in our models were normally distributed. One outlier, defined as any data point more than 3 times the interquartile range above the third or below the first quartile, was identified in our data. The outlier in question pertained to internal detail production only. To ensure that results were not influenced by the outlier, we ran analyses with the data point excluded. The pattern of results did not change, and thus our results are reported with this memory included.

Consistent with our hypothesis, our main analysis revealed that the more own visual perspectives shifted over time, the less consistent memories were between sessions (β = −0.13, *p* = .004), accounting for 28.4% (95% CI = [.22, .35]) of the variance in consistency observed. Similarly, the more observer visual perspectives shifted over time, the less consistent memories were between sessions (β = −0.11, *p* = .017), accounting for 26.4% (95% CI = [.20, .33]) of the variance in consistency observed. Entering both own and observer perspective ratings into our model did not increase the variance explained, suggesting that shifts in either perspective predict a substantial portion of changes in memory consistency (see Table 2).

**Table 2.** Results of Mixed Linear Modeling (MLM) Consistency Analysis

Predictors	Episodic/internal-detail consistency			Event-detail consistency			Perceptual-detail consistency			Emotion/thought-detail consistency		
	β	*p*	*R* ^2^	β	*p*	*R* ^2^	β	*p*	*R* ^2^	β	*p*	*R* ^2^
Own	−0.13	.004	.28	−0.08	.083	.24	0.02	.708	.07	−0.08	.106	.22
Observer	−0.11	.017	.26	−0.05	.239	.23	−0.06	.213	.07	−0.16	.001	.23
Own + Observer			.28			.24			.07			.23
Own	−0.10	.062		−0.07	.197		0.09	.152		0.02	.796	
Observer	−0.05	.385		−0.01	.841		−0.11	.062		−0.17	.006	

Note: Results of MLM analysis revealed that shifts in own and observer perspective independently predicted lower consistency of episodic details provided across sessions. Including both perspectives (i.e., Own + Observer) in the same model did not account for more variance. Furthermore, although shifts in own perspective were not driven by a specific AI detail subtype, the relationship between shifts in observer perspective and lower consistency was associated with a lack of consistency in emotion/thought details per se.

Follow-up exploratory analyses were run to examine whether direction of shifts in visual perspective was related to consistency by using difference scores in place of absolute-value shifts in perspective ratings. Results of follow-up analyses were run after our main analyses. These effects were nonsignificant, indicating that a change, more so than a loss or gain, in a given perspective predicted the consistency of the memory. Furthermore, as memories have been found to stabilize over time (e.g., Winningham et al., 2000; also see Bauer, 2015), and our initial retrieval window spanned 1 to 21 days, we conducted a sensitivity analysis that included the age of the event at Session 1 in our models to ensure that any relationship observed between consistency and visual perspective was not attributable to time. The pattern of results did not change (see Table S6 in the Supplemental Material).

**Fig. 3.**
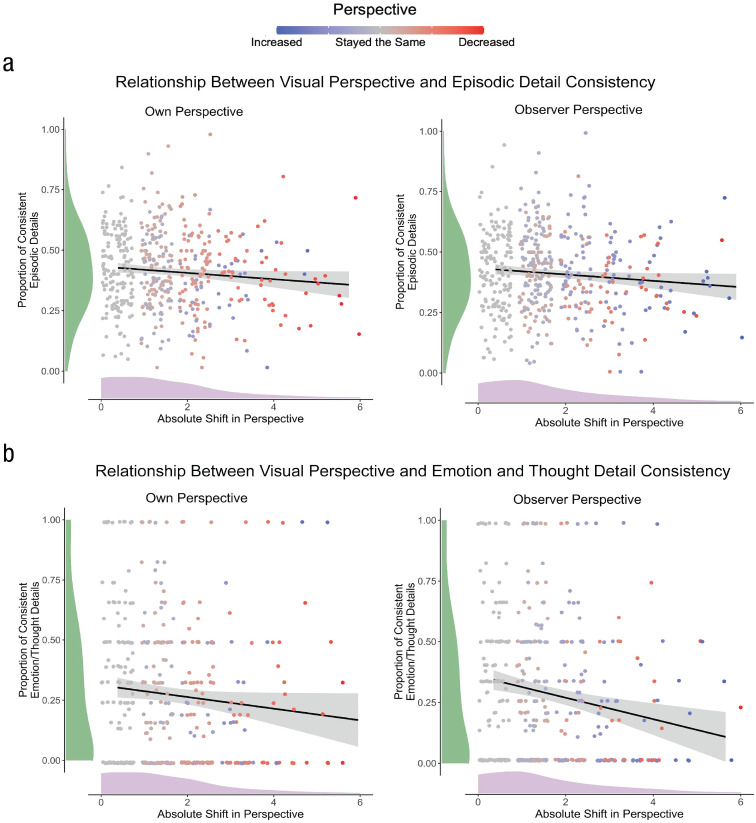
Shifts in visual perspective predict consistency of recalls. Greater changes in own and observer visual perspective predicted lower consistency of memories at Session 2 (a). Furthermore, the relationship between consistency and own perspective was not driven by a specific detail type. In contrast, the relationship between consistency and observer visual perspective was driven by changes in the consistency of emotion/thought details provided across sessions per se (b). Shifts in perspective ratings were calculated by computing difference scores (absolute value) between ratings at Session 1 and Session 2. Data were analyzed using mixed linear modeling (see the main text for details). Violin plots along x-axes represent distribution of absolute value shifts in perspective ratings and violin plots along y-axes represent distribution of consistent details. Plotted black lines reflects the linear relationship between perspective shift and consistent details, with grey error bands reflecting 95% confidence intervals.

We then turned to individual AI detail subtypes to assess whether the relationship observed between shifts in visual perspectives and changes in memory consistency were driven by changes in consistency associated with a specific detail subtype. We restricted our analyses to event, perceptual, and emotion/thought details because the range of place (0–14; *Mdn* = 2; *M* = 2.13, *SD* = 2.12) and time (0–8; *Mdn* = 0; *M* = 0.76, *SD* = 1.03) details provided at Session 2 was restricted. For shifts in own perspective, no significant effects on memory consistency were observed for specific AI detail subtypes. In contrast, shifts in observer perspective were specifically associated with the consistency of emotion/thought details (β = −0.16, *p* = .001; *R*^2^ = .23, 95% CI = [.16, .29]); that is, the greater the shift in observer perspective, the more inconsistent participants were for emotion/thought details. No significant effects were observed for the remaining AI subdetail types (all *p*s > .083). See Figure 3 for visualizations of significant effects.

## Discussion

We found that naturally occurring shifts in visual perspective are associated with changes in the consistency of voluntary autobiographical memories; less consistent recalls were associated with larger shifts in both own and observer perspectives. Although the relationship between consistency and shifts in own perspective was not driven by a particular type of detail, shifts in observer perspective were associated with less consistent emotion/thought details. We first discuss the phenomenon of visual perspective change in its own right and then discuss its relationship with memory consistency.

Visual perspectives underwent large shifts over 10 weeks, with similar absolute-value changes for both own (18.7% shift from Session 1) and observer (19.7% shift from Session 1) perspectives. On average, we observed a decrease in own and an increase in observer perspective, akin to findings observed in retrospective and cross-sectional studies (e.g., Rice & Rubin, 2009) and work that has measured own and observer perspectives on a single scale (Talarico & Rubin, 2003). Yet there was considerable variability in the direction of shifts in our data. Certain event characteristics may predict perspective changes given that some types of events seem to encourage a given perspective over another—events involving self-evaluation versus evaluation of others elicit more observer than own perspective, and vice versa (D’Argembeau & Van der Linden, 2008; also see Rice & Rubin, 2011). Alternatively, degree and direction of perspective shifts may reflect individual differences (Berg et al., 2021; Rubin, 2021). Future work exploring these possibilities is important.

Shifts in both own and observer perspective predicted lower consistency in the episodic content recollected over time, suggesting that changes in perspective represent changes in memory. The mental imagery that typically accompanies recall likely mimics memory’s reconstructive nature (Moscovitch, 2008; Schacter et al., 2011). Here, we use the term *reconstruction* broadly to refer to the process of piecing together elements of past experiences to be recalled in the present moment. Notably, memory retrieval varies in the intentionality and effort involved (see Barzykowski & Staugaard, 2016). Some researchers have posited that different pathways to retrieval may be driven by distinct types of reconstruction (see Harris et al., 2015). Because past research has shown that direct manipulation of perspective can alter memories, this relationship may be bidirectional. For example, Marcotti and St. Jacques (2018) found that adopting an observer perspective when recalling a laboratory experience leads to a reduction in overall accuracy for temporal order, spatial relations, actions, and sensations. In another study, memory accuracy for spatial, but not nonspatial, details was lower when individuals reviewed event photographs from an observer perspective before recall (Marcotti & St. Jacques, 2022). The limited range of time and space details recalled in our data precluded analysis of these detail types.

Instead, we found that shifts in observer perspective were particularly associated with reductions in the consistency of emotion/thought details, indicating that observer perspective might reflect an ability to change one’s internal experience of an event after it has occurred. Shifting to an observer perspective can impact the emotionality of memories (Küçüktaş & St. Jacques, 2022), perhaps allowing us to distance ourselves from the past so that we can remember events without reexperiencing every detail (Fernández, 2015; Libby & Eibach, 2011; McIsaac & Eich, 2004; Siedlecki, 2015). Indeed, observer perspectives are more likely to accompany recollection of events that elicit high degrees of self-awareness or distress (D’Argembeau & Van der Linden, 2008; Rice & Rubin, 2011). In light of our findings, we looked at the relationship between shifts in observer perspective and ratings of emotional valence and arousal. We found no relationship (all *p*s > .05). That our data show a relationship between observer perspective and emotion/thought details for memories of everyday experiences suggests that the utility of this mechanism may go beyond distancing the self from uncomfortable moments. Perhaps observer perspectives allow us to experience the event as someone other than ourselves—literally enabling us to adopt another’s point of view. Although the idea of a “social perspective” has not been explored in depth, observer perspective may be used in the service of understanding others’ event interpretations (Libby & Eibach, 2011). A related idea is that as memories age, one’s sense of self in a memory changes (i.e., that was “past me”). Shifts up or down in observer perspective may reflect changes in the degree to which one toggles between an emphasis on the perspective of different versions of the self and others.

In the spirit of observing memories naturalistically, we opted for a correlational approach. Thus, we cannot ascertain whether changes in perspective are causally related to changes in consistency. Moreover, consistency is not synonymous with accuracy; we cannot verify details. Still, our findings suggest that memories that veer from their original perspective are not necessarily less trustworthy with respect to the unfolding of the event or the perceptual content, a finding of particular importance in eyewitness testimony. Integrating this study with work that has manipulated perspective (e.g., Marcotti & St. Jacques, 2018) suggests that the relationship between perspective and memory fidelity is nuanced and divergent in naturalistic versus laboratory settings. Still, it is not possible to predict whether the effects observed here would be present for the types of events that are common subjects in such contexts. Unlike the courtroom, where high fidelity is critical, clinical work targeting appraisals of past experiences may benefit from encouraging shifts in observer perspective, given the relationship observed between observer perspective and malleability in emotion/thought details. Exploring memory phenomenology in therapeutic techniques such as emotion regulation (e.g., Webb et al., 2012) and resolving past experiences (i.e., “closure”; see Crawley, 2010) will be important to consider in light of the present findings and clinical work implicating mental imagery as a powerful therapeutic tool (Blackwell, 2019; Hackmann & Holmes, 2004). Still, further work is needed to understand whether the relationship between changes in emotion/thought details and observer perspective is causal and whether such a relationship may be similar or different for more emotionally evocative or traumatic events (see Berntsen & Nielsen, 2022; Talarico & Rubin, 2003). As recall of emotion/thought details has been found to distinguish emotional from neutral events in naturalistic narrative recall (see St. Jacques & Levine, 2007; Wardell, Madan, et al., 2021), exploring nuances in the relationship between detail consistency, perspective, and emotion in autobiographical memories are exciting avenues for future research. Indeed, some evidence indicates that voluntary autobiographical memories are more likely to be associated with observer than own perspectives in some clinical populations, including those with depression (Kuyken & Moulds, 2009; Warne & Rice, 2022) and posttraumatic stress disorder (Berntsen et al., 2003). Understanding the timing of shifts toward observer perspectives, and whether the shift coincides, drives, or follows changes in consistency, will be important to explore in order to bridge the current work with these clinical data.

Importantly, autobiographical memories are informed not only by the content we are attempting to remember but also by retrieval context and demands—the intention and utility of remembering shapes how the memory manifests (Barzykowski et al., 2023; Barzykowski & Mazzoni, 2022; Harris et al., 2015). Here, we focus on everyday, voluntarily recalled autobiographical memories that were retrieved following directed instructions targeting specific past episodes. Future work exploring the relationship between perspective and consistency in involuntary autobiographical memories, autobiographical memory at varying levels of episodicity, and effort or mode of retrieval (e.g., direct vs. generative) will be crucial in identifying the boundaries of the relationship demonstrated here. Probing memories at varied delays will also be important to explore: Here, we initially collected memories of events that occurred 1 to 24 days ago and again after a retention interval of approximately 10 weeks. Shifting these intervals will be crucial in developing our understanding of the life course of an autobiographical memory. Further, demonstration of the relationship between visual perspective and memory consistency in more diverse, community samples with balanced gender ratios is needed to generalize these findings more broadly.

Memories provide us with the record of our past. Yet the reconstructive nature of memory can render this record labile and, at times, misleading. Even the most faithful memories are reconstructions. The ability for humans to change the perspective of a memory in the mind’s eye, be it from an own or an observer perspective, mirrors memory’s reconstructive nature. We show that shifts in perspective over time predict the consistency of episodic recall. Memory for the emotions/thoughts experienced is particularly vulnerable—or apt—to change in relation to perspective. Humans can take varied perspectives, which may offer us a unique social advantage, allowing us to step into the eyes of another or a different version of ourselves. The usefulness of this feat may outweigh the cost to memory fidelity. However, such findings ask us to reconsider how to understand our memories, as there may be a need to shift away from an emphasis on reality and embrace our ability to retroactively adjust our experiences.

## Supplemental Material

sj-docx-1-pss-10.1177_09567976231180588 – Supplemental material for Fade In, Fade Out: Do Shifts in Visual Perspective Predict the Consistency of Real-World Memories? Supplemental material, sj-docx-1-pss-10.1177_09567976231180588 for Fade In, Fade Out: Do Shifts in Visual Perspective Predict the Consistency of Real-World Memories? by Victoria Wardell, Taylyn Jameson, Oliver J. R. Bontkes, M. Lindy Le, Tz-yu Duan, Peggy L. St. Jacques, Christopher R. Madan and Daniela J. Palombo in Psychological Science

## Transparency

*Action Editor:* Karen Siedlecki

*Editor:* Patricia J. Bauer

*Author Contributions*

**Victoria Wardell:** Conceptualization; Data curation; Formal analysis; Investigation; Methodology; Project administration; Validation; Visualization; Writing – original draft; Writing – review & editing.

**Taylyn Jameson:** Conceptualization; Data curation; Investigation; Methodology; Writing – review & editing.

**Oliver J. R. Bontkes:** Investigation; Validation; Writing – review & editing.

**M. Lindy Le:** Investigation; Writing – review & editing.

**Tz-yu Duan:** Investigation; Writing – review & editing.

**Peggy L. St. Jacques:** Methodology; Writing – review & editing.

**Christopher R. Madan:** Methodology; Validation; Writing – review & editing.

**Daniela J. Palombo:** Conceptualization; Funding acquisition; Methodology; Project administration; Resources; Validation; Writing – review & editing.

## Notes

1. AI details are, in part, influenced by the narrative structure of the detail provided. For example, “the lights were on” would be scored as a perceptual detail, whereas “I turned the lights on” would be scored as an event detail. Our AI-CONS scoring procedure does not penalize participants for recalling consistent details framed in ways that shift AI detail categorization across sessions, despite introducing a small amount of noise into the data (e.g., the proportion of consistent event details provided at Session 2 and the proportion of omitted event details provided at Session 1 will, in a small minority of memories, not equal one).
