# Supplementary material for: Fade In, Fade Out: Do Shifts in Visual Perspective Predict the Consistency of Real-World Memories?
Source: Psychol Sci. 2023 Jul 13;34(8):932–46. doi: 10.1177/09567976231180588 (PMC13038080; doi:10.1177/09567976231180588)
Supplement: sj-docx-1-pss-10.1177_09567976231180588 – Supplemental material for Fade In, Fade Out: Do Shifts in Visual Perspective Predict the Consistency of Real-World Memories? [file sj-docx-1-pss-10.1177_09567976231180588.docx]

**Supplemental Materials**

**Event Selection Instructions.** The following instructions were provided to participants during event selection.

Please come up with personal events from your life that you are comfortable sharing. These events should be from within the past two weeks, but should not include events that occurred today.

*Please do...*

Select events that you were personally involved in, and have a recollection of being personally involved in. The events must be from a specific time and place, typically lasting no more than a few hours. For example, describing a weekend trip to your cabin would not be sufficient. However, a specific incident that happened over that weekend, such as going water skiing with your friend, would be good. Try to come up with six unique events.

*Please do not...*

Select events that have similar themes, for example, not all of your memories should be of a camping trip. Please do not include overly mundane events, or things you do often, such as “making myself breakfast”, etc. Please do not include events in which you were under the influence of substances, such as alcohol or drugs. Please avoid extremely emotional events, including traumatic experiences. Please do not include virtual meetings or "hang-outs" such as classes held over Zoom or Facetiming with a friend. Finally, please do not include academic course related events, such as taking a test or exam, or completing an assignment.

Please select and title your 6 events. Please include a description of:

**What** happened. For example, a good "what" would be "Going on a 30-minute bike ride with Jane." or "Watching the first Harry Potter movie". On the other hand, something like "Bike ride" or "Watching a movie" is insufficient.

**Where** it happened. For example, a good "where" would be "Arbutus Greenway." or "Bean Around the World". On the other hand, something like "Bike path" or "Coffee shop" is insufficient.

**When** it happened (i.e., 2020/10/21, in yyyy/mm/dd format). We recognize you might not know the exact date that an event occurred. In this case, please take your best guess and enter an approximate date. Please do not refer to a calendar.

We ask that your event title be specific enough that by reading the title you will know exactly which event it refers to.

**Definition of Own and Observer Perspectives.** The following description of visual perspectives was provided to participants when they were asked to rate the perspective from which they pictured their memories.

When people recall an event from their past, often the memory triggers imagery within the “mind's eye”. This image is pictured from a visual perspective, that is, we view the event from a specific point of view, either from our own perspective, an observer's perspective, or a mix of both. When we picture a past event from our own perspective, we see it from our point of view, in first-person, as we would have seen it during the actual event. We are in our own bodies and watching the event unfold. In contrast, when we picture a past event from an observer's perspective, we see it in third-person, as if we were watching ourselves in the event, perhaps from a birds-eye view or similar outsider point of view. For example, when remembering a speech or presentation, you might have a picture of standing behind a podium, watching the audience as you speak. This would be from your own perspective. Alternatively, you might picture the event as if you were an audience member, watching yourself stand there and present. This would be from an observer's perspective.

**Table S1.** *Self-Reported Phenomenological Characteristics*

| **Construct** | **Item** | **Source** |
| --- | --- | --- |
| Observer Perspective | To what degree is the memory you have for this event pictured from an observer's perspective?  *1 (No imagery in my mind's eye is from an observer's perspective) –*  *7 (All the imagery in my mind's eye is from an observer's perspective)* | See Rice & Rubin, 2009 |
| Own Perspective | To what degree is the memory you have for this event pictured from your own perspective?  *1 (No imagery in my mind's eye is from my own perspective) –*  *7 (All the imagery in my mind's eye is from my own perspective)* |  |
| Vividness | While remembering the event, I feel as though I am reliving it.  *1 (Not at all) – 7 (As clearly as if it were happening now)* | Talarico et al., 2004 |
|  | While remembering the event, I can see it in my mind.  *1 (Not at all) – 7 (As clearly as if it were happening now)* |  |
| Belief | I believe the event in my memory really occurred in the way I remember it and that I have not imagined or fabricated anything that did not occur.  *1 (100% imaginary) – 7 (100% real)* |  |
| Rehearsal | Since it happened, I have thought or talked about this event.  *1 (Not at all) – 7 (More than for any other memory)* |  |
| Coherence | My memory comes to me in words or in pictures as a coherent story or episode and not as an isolated fact, observation, or scene.  *1 (Not at all) – 7 (Completely)* |  |
|  | This memory comes in pieces, with bits missing.  *1 (Not at all) – 7 (Completely)* |  |
| Importance Now | How personally important IS this event to you NOW?  *1 (No importance at all) – 6 (Of great importance)* | Levine et al., 2002 |
| Importance Then | How personally important WAS this event to you THEN?  *1 (No importance at all) – 6 (Of great importance)* |  |
| Emotional Valence | How emotional was this event? Memories with close to no emotional content should be rated -1 or 1  *-5 (Intense negative emotion) – 5 (Intense positive emotion)* | Wardell et al., 2021 |
| Arousal | How did you feel during this event?  *1 (Very calm) – 6 (Very aroused (excited or agitated))* |  |
| Uniqueness | How unique was this event?  *1 (Something I do very often) – 6 (Something I rarely do)* |  |

*Note.* Self-report ratings for phenomenological characteristics of the memory were obtained for events at both session one and session two. Vividness and coherence scores were calculated by averaging ratings on the two items associated with the construct.

**Recall Instructions.** The following instructions and example of a memory recall were provided to participants to ensure they understood the types of details we were asking them to provide.

In the next section, you will be asked to type out everything that you can remember about three of these specific events. Please type out all details that come to mind for each specific event. Nothing is too trivial. This exercise is meant to be a stream of consciousness. Please do not worry about grammar, spelling, or flow. This is not an essay. Please type everything that comes to mind. To ensure you have provided as many details as possible, you will not be able to move forward until you have entered at least ~300 words (1200 characters). Please read through the example below to get a sense of what we are looking for.

“It was my graduation ceremony for my PhD. Before my graduation ceremony I decided to go to the mall with my sister to kill some time before the ceremony and so we went to Sherway, and while we were there at some point, just as I was gonna leave, I ran into my friend. And her mom. And it was a good friend that I haven't seen in a long time. So, even though I felt like I really needed to go to get to the ceremony I decided to stay a few extra minutes to talk to her. And then I realized I really had to leave, and I got into the car and I started to feel a little bit nervous that I was running late. But I got in the car, and I went on the highway, and there was a lot of traffic. So I started to get even more nervous that I was gonna be late. And I didn't have a cell phone at the time, so I couldn’t call anyone that was gonna be at the ceremony to let them know that I was late. So my husband and my parents and my sister were meeting me at the Convocation Hall. So eventually I got there with a couple minutes left to spare. But realized that I need to find parking. And everything was super busy, there were no parking spots anywhere near Convocation Hall so I had to drive around and was getting more and more anxious. And feeling like I was really running out of time. I eventually found a parking spot and ran over to the front of Convocation Hall where I saw my husband and my family looking at me like, where have you been? And feeling like I was gonna cry. And somebody came up to me and helped me, sort of, get sorted out with my cap and gown. And I was able to get that just moments before my class was walking in. And so I snuck to the back of the line and managed to make it inside on time. And I remember after the ceremony being outside taking pictures with my husband and sister and parents. And my mom was wearing a white jacket and my husband was wearing a suit.”

**Table S2.** *Questionnaire Battery*

| **Construct** | **Questionnaire** | **Source** | **Session Collected** |
| --- | --- | --- | --- |
| Depression | Center for Epidemiological Studies-Depression (CES-D) | Radloff, 1977 | 1 and 2 |
| Anxiety | Shortened State/Trait Anxiety Inventory (STAI) | Zsido et al., 2020 | 1 and 2 |
| Rumination | Ruminative Thought Style Questionnaire (RTS) | Brinker & Dozois, 2009 | 1 |
| Dissociation | Dissociative Experiences Scale (DES-II) | Carlson & Putnam, 1993 | 1 |
| Verbal Ability | Shipley-2 | Shipley et al., 2009 | 1 |
| Divergent Thinking/  Creativity | Alternative Uses Task (AUT) | Silvia et al., 2008 | 2 |
| Mental Imagery | Object-Spatial Imagery Questionnaire (OSIQ) | Blajenkova et al., 2006 | 2 |

*Note.* Data from questionnaires was collected for ancillary hypotheses beyond the scope of the present paper and are not reported here.

**Scored Recall.** The following example reflects a typical recall and the scoring procedures we applied. We first scored recalls in accordance with Levine and colleagues' Autobiographical Interview (AI; 2002). While our analysis concerned total internal details provided (i.e., episodic details specific to the event being recalled), we scored external details (i.e., details non-specific to the event being recalled) as well as the sub-details of internal and external detail categories as per the AI protocol.

**
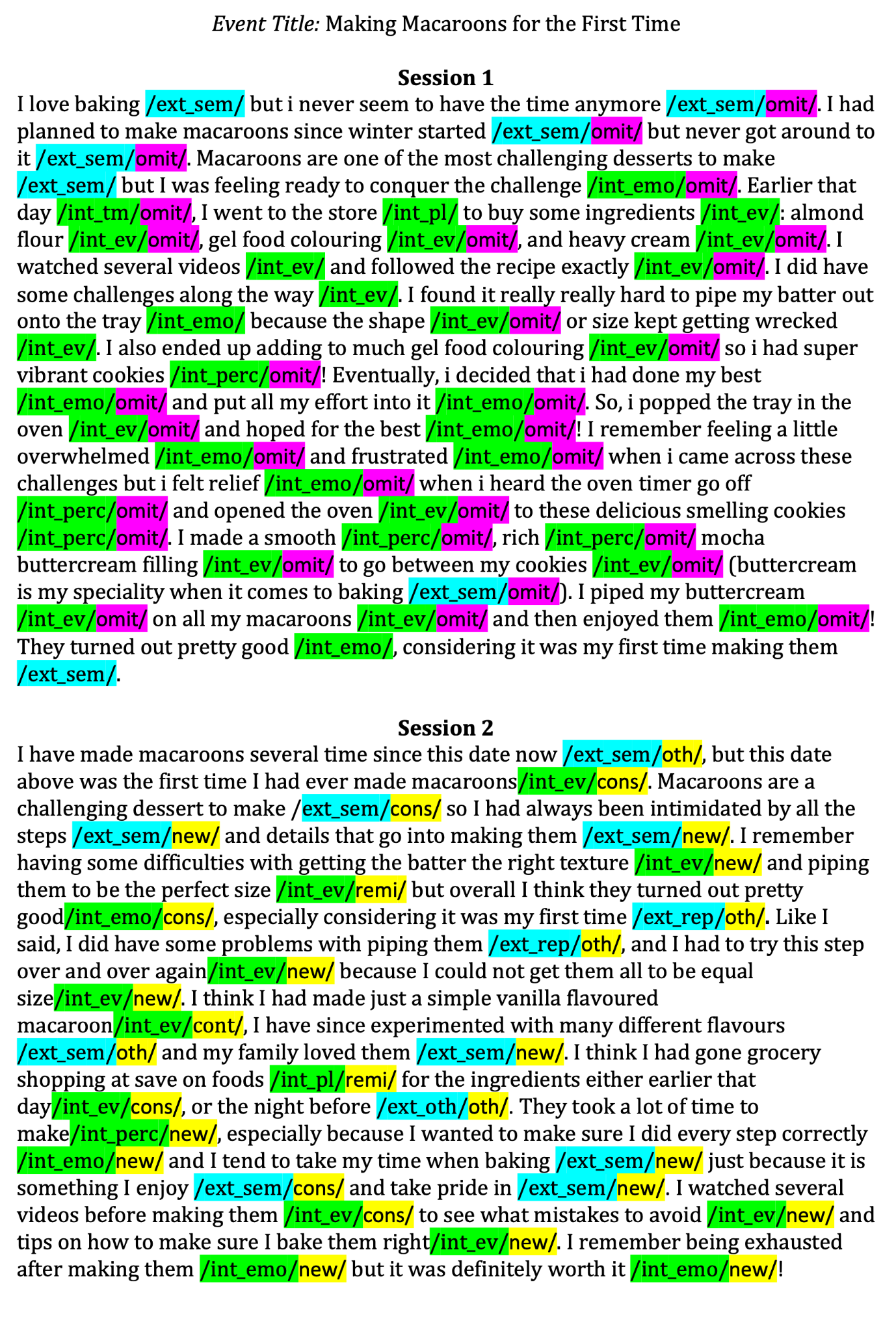
**

*Note.* AI Details are coded in green (internal details, i.e., ‘int’) and blue (external details, i.e., ‘ext’), where tm = time, pl = place, ev = event, perc = perceptual, emo = emotion/thought, sem = semantic, oth = other, rep = repetition. AI-CONS details are coded in pink (session one consistency) and yellow (session two consistency), where omit = omitted, oth = other, cons = consistent, new = new, remi = reminiscent.

**Table S3.** *Cronbach’s Alpha Scores for Scored Detail*

| Category | | Detail Type | ICC | Fixed Effects | *p*-value | 95% CI |
| --- | --- | --- | --- | --- | --- | --- |
| AI | Internal | Total | .97 | *F_(91, 273)_* = 38.0 | < .001 | [.96, .98] |
|  |  | Event | .95 | *F_(91, 273)_* = 22.0 | < .001 | [.94, .97] |
|  |  | Perception | .92 | *F_(91, 273)_* = 13.3 | *<* .001 | [.90, .95] |
|  |  | Emotion/Thought | .92 | *F_(91, 273)_* = 13.0 | < .001 | [.89, .95] |
|  |  | Place | .93 | *F_(91, 273)_* = 15.0 | < .001 | [.91, .95] |
|  |  | Time | .94 | *F_(91, 273)_* = 18.0 | < .001 | [.92, .96] |
|  | External | Total | .93 | *F_(91, 273)_* = 14.0 | < .001 | [.90, .95] |
|  |  | Semantic | .94 | *F_(91, 273)_* = 18.0 | < .001 | [.92, .96] |
|  |  | Extraneous Event | .80 | *F_(91, 273)_* = 5.1 | < .001 | [.73, .86] |
|  |  | Repetition | .55 | *F_(91, 273)_* = 2.2 | < .001 | [.38, .68] |
|  |  | Other | .90 | *F_(91, 273)_* = 9.8 | < .001 | [.86, .93] |
| AI-CONS | | Consistent | .94 | *F_(46. 46)_* = 17.0 | < .001 | [.89, .97] |
|  |  | Contradictory | .79 | *F_(46. 46)_* = 4.8 | < .001 | [.62, .88] |
|  |  | Reminiscent | .51 | *F_(46. 46)_* = 2.0 | .008 | [.12, .73] |
|  |  | Other | .98 | *F_(46. 46)_* = 58.0 | < .001 | [.97, .99] |
|  |  | New | .96 | *F_(46. 46)_* = 23.0 | < .001 | [.92, .98] |
|  |  | Omitted | .97 | *F_(46. 46)_* = 40.0 | < .001 | [.96, .99] |

*Note.* Inter-rater reliability for each detail type, with raters as fixed effects. We note that poor reliability for repetition details in AI scoring as well as contradictory and reminiscent details in consistency scoring were a result of encountering a floor effect for these detail types, with 98.5% of memories containing two or fewer repetition details, 82.8% of session 2 memories containing two or fewer contradictory details, and 82.6% of session 2 memories containing two or fewer reminiscent details. Neither detail type was used in any of our analyses.

**Table S4.** *Descriptive Statistics of Phenomenological Characteristic Ratings*

|  | Recalled Memories  (*N* = 470) | | Not-Recalled Memories  (*N* = 484) | |
| --- | --- | --- | --- | --- |
|  | S1 | S2 | S1 | S2 |
|  | *M*(*SD*) | *M*(*SD*) | *M*(*SD*) | *M*(*SD*) |
| Own Perspective | 5.56(1.48) | 5.01(1.61) | 5.54(1.49) | 5.00(1.60) |
| Observer Perspective | 2.67(1.63) | 3.02(1.68) | 2.57(1.62) | 2.93(1.65) |
| Emotional Valence | 1.84(1.98) | 1.75(2.03) | 1.79(2.01) | 1.76(1.83) |
| Arousal | 3.45(1.65) | 3.50(1.44) | 3.35(1.52) | 3.50(1.42) |
| Importance Now | 2.97(1.46) | 2.60(1.48) | 2.96(1.43) | 2.52(1.39) |
| Importance Then | 3.62(1.50) | 3.59(1.42) | 3.60(1.48) | 3.50(1.45) |
| Uniqueness | 3.99(1.68) | 3.89(1.63) | 3.92(1.63) | 3.84(1.67) |
| Belief in Accuracy | 6.14(1.12) | 5.42(1.38) | 6.15(1.14) | 5.37(1.44) |
| Vividness | 4.63(1.33) | 4.01(1.40) | 4.67(1.39) | 3.90(1.36) |
| Rehearsal | 2.82(1.38) | 2.27(1.29) | 2.86(1.47) | 2.18(1.28) |
| Coherence | 4.48(1.51) | 3.62(1.47) | 4.41(1.54) | 3.60(1.52) |
| Event Age | 7.08(4.21) | 84.44(5.15) | 6.58(4.20) | 84.02(5.34) |
| Retention Interval | – | 77.36(3.15) | – | 77.45(3.25) |

*Note.* A total of 178 participants provided 470 recalled events. Not-recalled events were subject to the same exclusion procedure as recalled events, save for recall quality checks. This led to the exclusion of 47 not-recalled events (32 for being outside of our three-week timeframe, 15 for not being remembered at session two). One participant had all three of their not-recalled events excluded. This resulted in a total of 177 participants providing 484 not-recalled events.

**Table S5.** *Differences in Absolute Value Shifts in Phenomenological Characteristics between Recalled and Not-Recalled Events*

|  | Recalled Events  (*N* = 177) | Not-Recalled Events  (*N* = 177) |  |  |
| --- | --- | --- | --- | --- |
|  | *M*(*SD*) | *M*(*SD*) | *t* | *p* |
| Own Perspective | 1.31(0.93) | 1.36(0.94) | 0.58 | .564 |
| Observer Perspective | 1.38(0.99) | 1.38(0.93) | 0.04 | .965 |
| Emotional Valence | 1.13(0.96) | 1.08(0.80) | 0.56 | .580 |
| Arousal | 1.05(0.70) | 1.06(0.72) | 0.16 | .875 |
| Importance Now | 1.00(0.62) | 0.96(0.68) | 0.62 | .534 |
| Importance Then | 0.90(0.57) | 0.89(0.54) | 0.09 | .930 |
| Uniqueness | 0.95(0.62) | 0.96(0.58) | 0.20 | .839 |
| Belief in Accuracy | 1.11(0.85) | 1.14(0.84) | 0.47 | .643 |
| Vividness | 1.18(0.71) | 1.26(0.75) | 1.36 | .175 |
| Rehearsal | 1.06(0.73) | 1.09(0.77) | 0.39 | .699 |
| Coherence | 1.45(0.88) | 1.35(0.81) | 1.31 | .191 |
| Event Age Session 1* | 7.00(2.79) | 6.59(2.66) | 1.41 | .161 |

*Note.* Absolute values of difference scores were calculated across sessions for phenomenological ratings of each event. Absolute values were then averaged within participants to compare recalled events and not-recalled events. As one participant had all three of their not-recalled events excluded, analyses were run on the remaining 177 participants.

*Absolute value difference scores were used for all variables except event age at session one, which instead reflects the number of days between the event and session one.

**Figure S1.** *Histogram of Visual Perspectives*

**
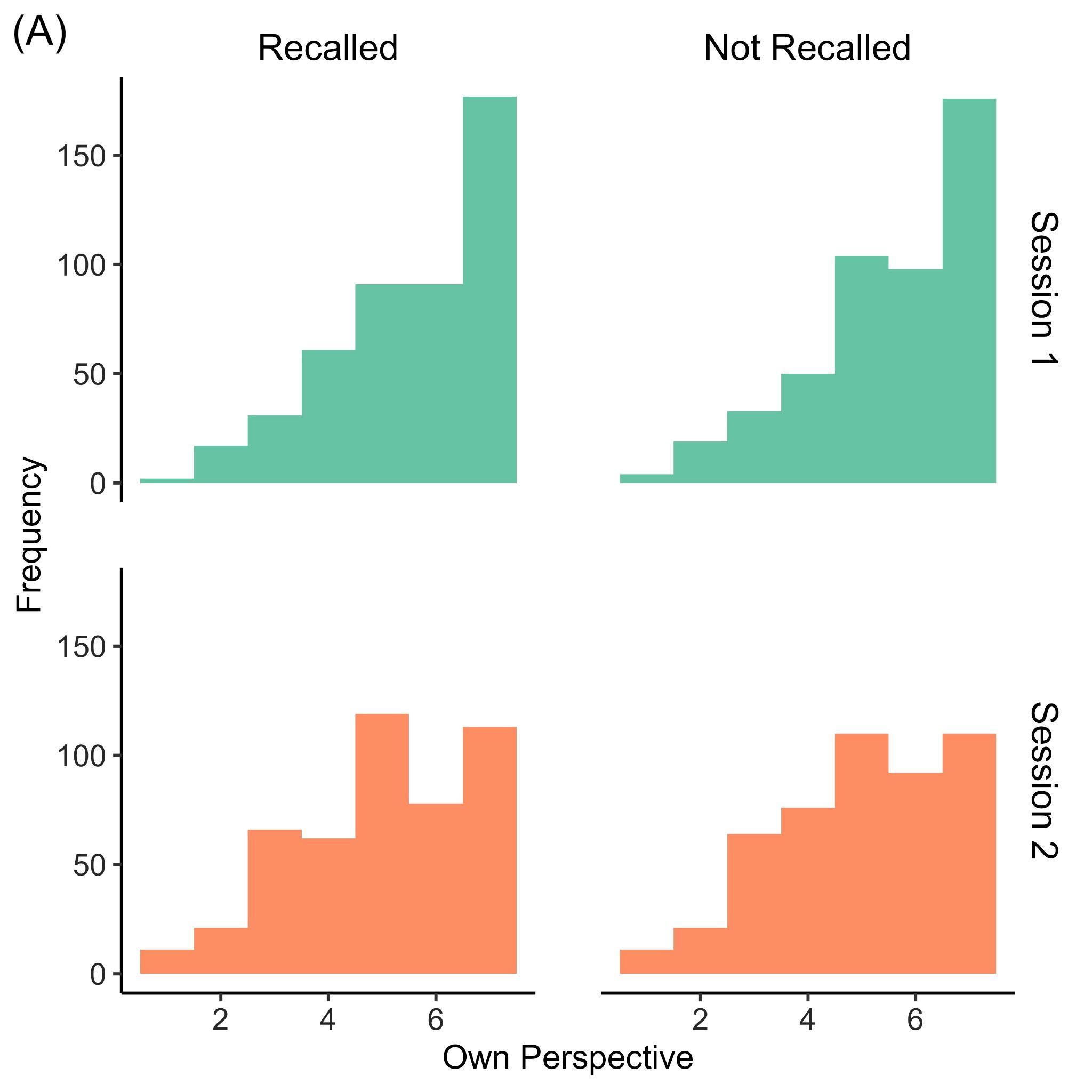

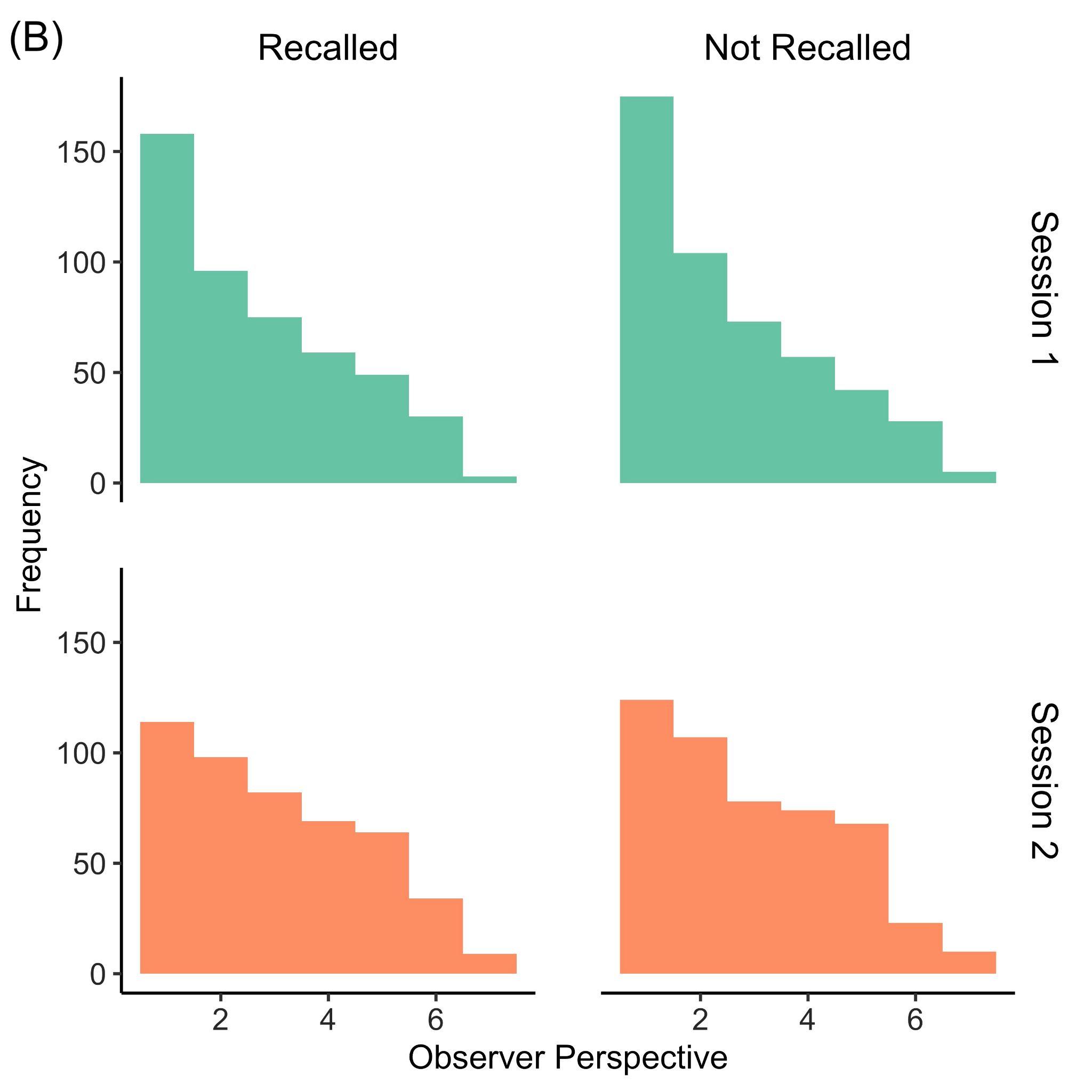
**

*Note.* Figure A depicts the distribution of own perspective for recalled and not recalled events by session. Figure B depicts the distribution of observer perspective for recalled and not recalled events by session.

**Table S6.** *Results of MLM when Controlling for Event Age at Session 1.*

|  | Episodic/Internal Detail Consistency | | | Event Detail Consistency | | | Perceptual Detail Consistency | | | Emotion/Thought Detail Consistency | | |
| --- | --- | --- | --- | --- | --- | --- | --- | --- | --- | --- | --- | --- |
|  | $\beta$ | *p* | *R^2^* | $\beta$ | *p* | *R^2^* | $\beta$ | *p* | *R^2^* | $\beta$ | *p* | *R^2^* |
| Own |  |  | 0.30 |  |  | 0.25 |  |  | 0.10 |  |  | 0.22 |
| Own | –.13 | .005 |  | –.07 | .098 |  | .02 | .645 |  | –.08 | .107 |  |
| Event Age | .15 | < .001 |  | .12 | .005 |  | .18 | < .001 |  | .002 | .964 |  |
| Observer |  |  | 0.28 |  |  | 0.24 |  |  | 0.11 |  |  | 0.22 |
| Observer | –.11 | .019 |  | –.05 | .262 |  | –.06 | .252 |  | –.16 | .001 |  |
| Event Age | .15 | < .001 |  | .13 | .005 |  | .17 | < .001 |  | –.0001 | .998 |  |
| Own + Observer |  |  | 0.30 |  |  | 0.25 |  |  | 0.11 |  |  | 0.22 |
| Own | –.10 | .071 |  | –.07 | .219 |  | .09 | .144 |  | .02 | .797 |  |
| Observer | –.05 | .388 |  | –.01 | .847 |  | –.11 | .071 |  | –.17 | .006 |  |
| Event Age | .15 | < .001 |  | .12 | .005 |  | .18 | < .001 |  | –.0001 | .999 |  |

*Note*. While event age at session one was related to the consistency of memories, controlling for event age in our model did not change the pattern of results observed between visual perspective and episodic detail consistency.

**References**

Blajenkova, O., Kozhevnikov, M., & Motes, M.A. (2006). Object-spatial imagery: A new self-report imagery questionnaire. *Applied Cognitive Psychology*, *20*, 239-263. <https://doi.org/10.1002/acp.1182>

Brinker, J. K., & Dozois, D. J. (2009). Ruminative thought style and depressed mood. *Journal of Clinical Psychology*, *65*(1), 1-19. <https://doi.org/10.1002/jclp.20542>

Carlson, E. B., & Putnam, F. W. (1993). An update on the Dissociative Experiences Scale. *Dissociation: Progress in the Dissociative Disorders, 6*(1), 16–27.

Levine, B., Svoboda, E., Hay, J. F., Winocur, G., & Moscovitch, M. (2002). Aging and autobiographical memory: Dissociating episodic from semantic retrieval. *Psychology and Aging, 17*(4), 677. <https://doi.org/10.1037/0882-7974.17.4.677>

Radloff, L. S. (1977). The CES-D scale: A self-report depression scale for research in the general population. *Applied Psychological Measurement*, *1*(3), 385-401. <https://doi.org/10.1177/014662167700100306>

Rice, H. J., & Rubin, D. C. (2009). I can see it both ways: First-and third-person visual perspectives at retrieval. *Consciousness & Cognition*, *18*(4), 877-890. <https://doi.org/10.1016/j.concog.2009.07.004>

Shipley, W. C., Gruber, C. P, Martin, T. A., & Klein, A. M. (2009). *Shipley-2 manual*. Western Psychological Services.

Silvia, P. J., Winterstein, B. P., Willse, J. T., Barona, C. M., Cram, J. T., Hess, K. I., Martinez, J. L., & Richard, C. A. (2008). Assessing creativity with divergent thinking tasks: Exploring the reliability and validity of new subjective scoring methods. *Psychology of Aesthetics, Creativity, and the Arts, 2*, 68-85. <https://doi.org/10.1037/1931-3896.2.2.68>

Talarico, J. M., LaBar, K. S., & Rubin, D. C. (2004). Emotional intensity predicts autobiographical memory experience. *Memory & Cognition*, *32*(7), 1118-1132. <https://doi.org/10.3758/BF03196886>

Wardell, V., Madan, C. R., Jameson, T. J., Cocquyt, C. M., Checknita, K., Liu, H., & Palombo, D. J. (2021). How emotion influences the details recalled in autobiographical memory. *Applied Cognitive Psychology, 35*(6), 1454-1465. <https://doi.org/10.1002/acp.3877>

Zsido, A. N., Teleki, S. A., Csokasi, K., Rozsa, S., & Bandi, S. A. (2020). Development of the short version of the Spielberger State—Trait Anxiety Inventory. *Psychiatry Research*, *291*, 113223. <https://doi.org/10.1016/j.psychres.2020.113223>
